# Supplementary material for: A fast-acting lipid checkpoint in G1 prevents mitotic defects
Source: Nat Commun. 2024 Mar 18;15:2441. doi: 10.1038/s41467-024-46696-9 (PMC10948896; doi:10.1038/s41467-024-46696-9)
Supplement: Supplementary file 1 — Supplementary Information [file 41467_2024_46696_MOESM1_ESM.pdf]

A fast-acting lipid checkpoint in G1 prevents mitotic defects

Marielle S. Köberlin[1,2] *, Yilin Fan[1,3], Chad Liu[1,4], Mingyu Chung[1,5], Antonio F. M. Pinto[6], Peter K. Jackson[2,7], Alan Saghatelian[6], Tobias Meyer[1,8] *

[1]Department of Chemical and Systems Biology, Stanford University School of Medicine, Stanford, CA 94305, USA

[2]Baxter Laboratory, Department of Microbiology & Immunology, Stanford University School of Medicine, Stanford, CA 94305, USA

[3]Current address: Department of Pathology and Center for Cancer Research, Massachusetts General Hospital and Harvard Medical School, Boston, MA 02114, USA

[4]Current Address: Chan Zuckerberg Biohub, San Francisco, CA 94111, USA

[5]Current Address: Department of Neurology and Neurological Sciences, Stanford University School of Medicine, Stanford, CA 94305, USA

[6]Clayton Foundation Laboratories for Peptide Biology and Mass Spectrometry Core, Salk Institute for Biological Studies, La Jolla, CA 92037, USA

[7]Department of Pathology, Stanford University School of Medicine, Stanford, CA 94305, USA

[8]Department of Cell and Developmental Biology, Weill Cornell Medicine, New York, NY 10065, USA

* For correspondence: mkoeberlin@stanford.edu (MSK), tom4003@med.cornell.edu (TM)

Supplementary File contains:

Supplementary Figures 1-7

Uncropped scans of Supplementary blots

Supplementary References

Supplementary Tables 1, 2

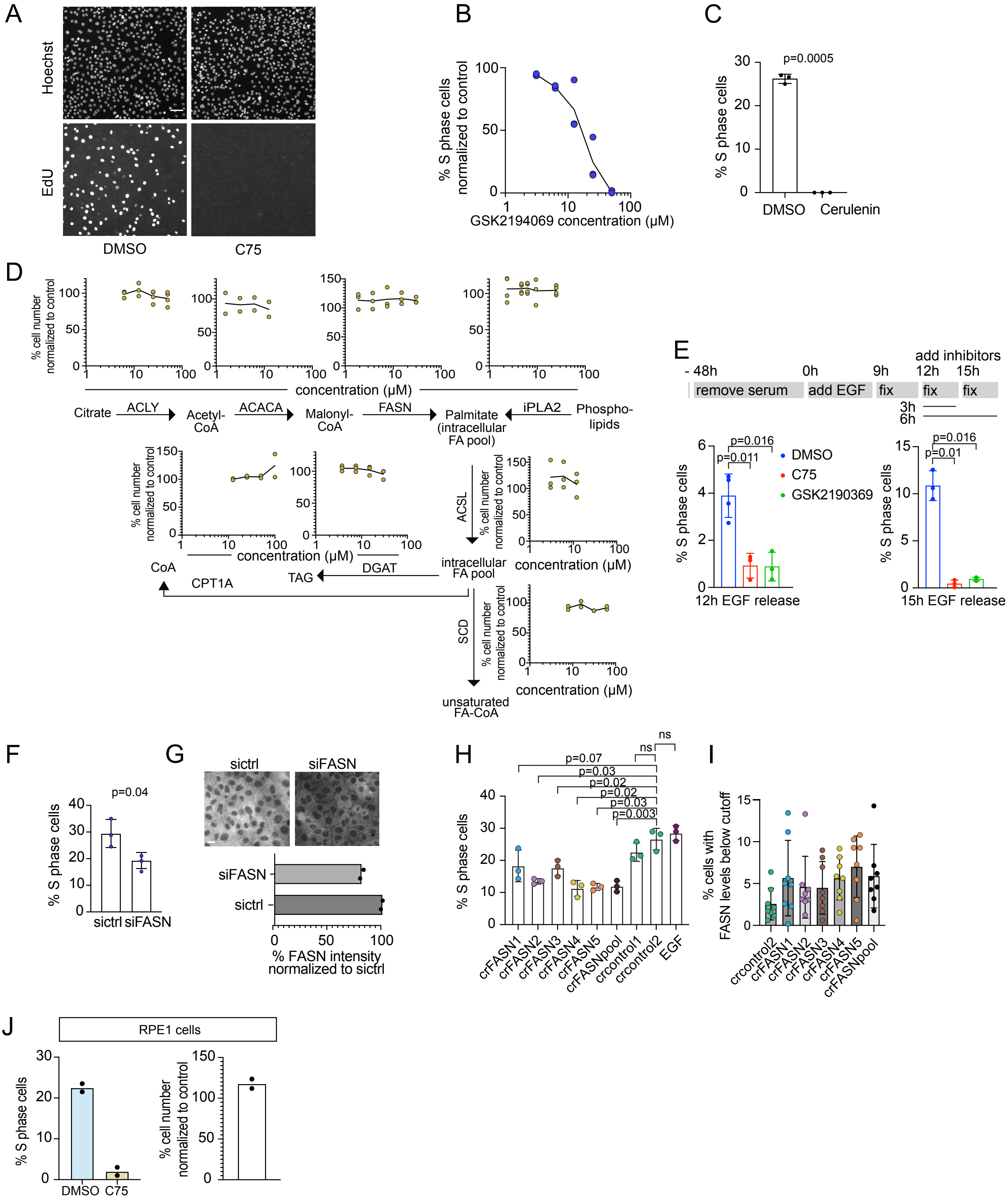

Fig. S1: FASN inhibition in G1 prevents S phase entry. (**A**) MCF-10A cells EGF-released and treated with DMSO or C75 (15µM). S phase cells are visualized by EdU signal, nuclei by Hoechst stain. Sample immunofluorescence images are shown. Scale bar: 10 µm. Data are representative of at least three independent experiments. (**B**) Percentage of EdU positive

cells treated with increasing concentrations of FASN inhibitor GSK2194069 and normalized to DMSO treatment. Data are from at least two independent experiments. (**C**) Percentage of EdU positive cells treated with DMSO or Cerulenin. Data are from three independent experiments, n > 23,000 cells per condition. (**D**) Percentage of EdU positive cells treated with increasing concentrations of fatty acid metabolism inhibitors and normalized to DMSO treatment. Metabolic pathway and targeted enzymes are shown. Data are from at least two independent experiments, n > 12,000 per condition. (**E**) Percentage of EdU positive cells 12 or 15 hours after EGF release. Cells were released for 9 hours and indicated inhibitors were added for 3 or 6 hours. Data are from three independent experiments, n > 14,000 cells per condition. (**F**) Percentage of EdU positive cells transfected with sicontrol (sictrl) or siFASN. Data are from three independent experiments, n > 21,000 cells per condition. (**G**) Staining of MCF-10A cells treated with sictrl or siFASN. Quantification of cytosolic FASN staining in siFASN treated cells normalized to sictrl. (**H**) Percentage of EdU positive cells in crFASN (individual and pooled crRNAs) and control (crcontrol1 and 2) treated MCF-10A-Cas9 cells. Data are from three independent experiments, n > 25,000 per condition. (**I**) Percentage of MCF-10A-Cas9 cells treated with individual crRNAs that are FASN negative (log_2 cutoff = 7) 68 hours after crRNA transfection based on immunofluorescence, n > 9,000 per condition (individual data points are percentages of cell population in 8 different regions imaged). (**J**) Percentage of EdU positive RPE1 cells 20 hours after release with growth media and 0.5% serum in the presence of DMSO or C75 (2μM) (left). Percentage of cell number after C75 treatment normalized to DMSO treated conditions (right). Data are from two independent experiments. (**C, E, F, H**) Mean and SD are shown. P values calculated using two-tailed paired t test. Unless indicated otherwise, EdU signal was measured 20 hours after EGF release. arb., arbitrary. Source data are provided as Source data file.

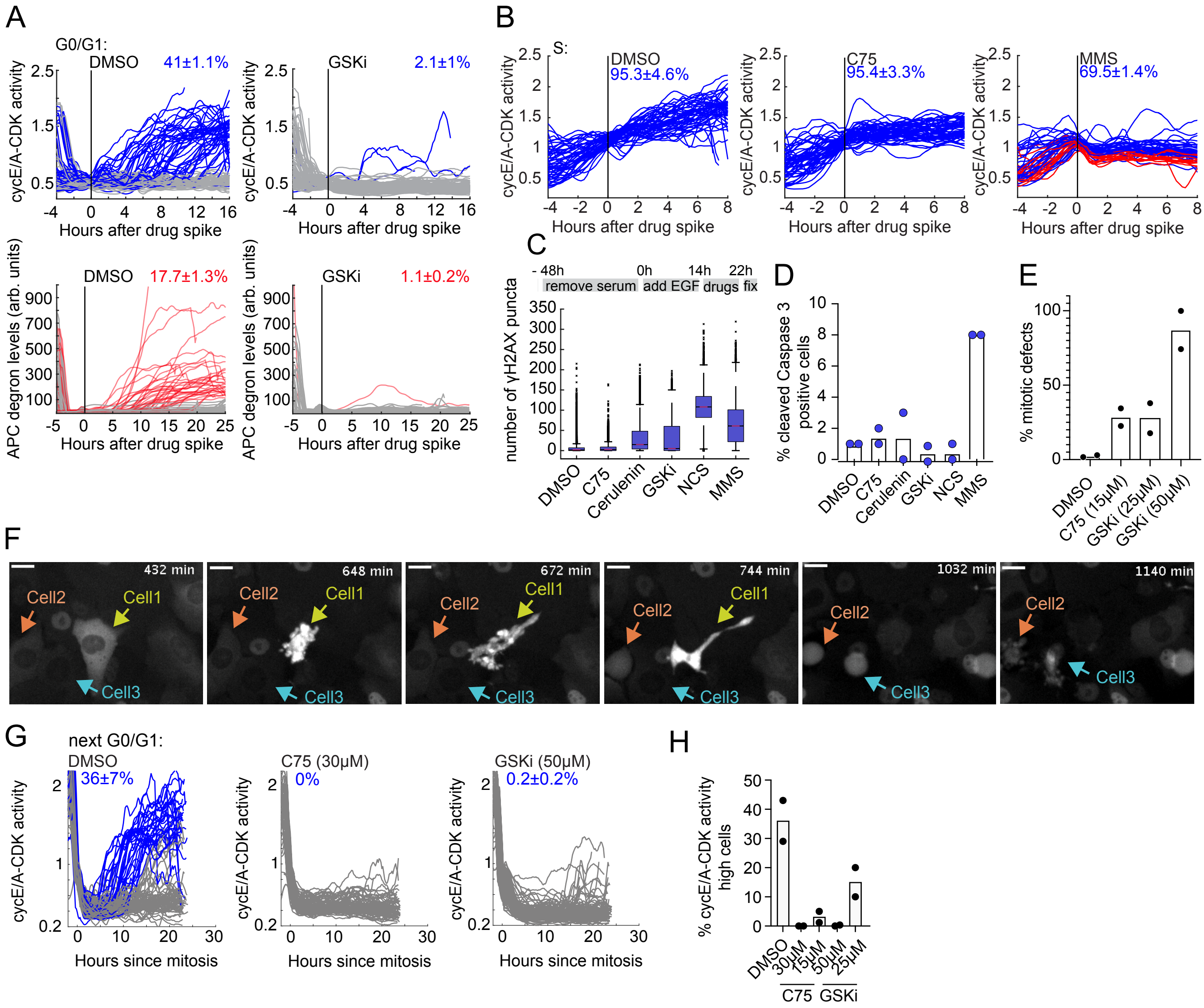

Fig. S2: FASN inhibition in G2/M causes mitotic defects. (**A**) Traces of cyclin-E/A CDK activity (blue) and APC/C^{Cdh1} degron reporter levels (red) in cycling MCF-10A cells treated with different inhibitors or DMSO during G0/G1 phase (cyclin-E/A CDK activity: 0.4-0.6 at the point of treatment). Top: Grey traces (future cyclin-E/A CDK activity < 1) and blue traces (> 1). Bottom: Red traces (future degron activity > 100) and grey traces (< 100). Quantification is percentage of cells with increased cyclin-E/A CDK activity (Top, blue) or increased APC degron levels (Bottom, red). (**B**) Traces of cyclin-E/A CDK activity (blue) in cells treated with different inhibitors or DMSO during S phase (cyclin-E/A CDK activity: 1-1.2 at the point of treatment). Red traces (cyclin-E/A CDK activity < 0.8). Quantification is percentage of cells with cyclin-E/A CDK activity > 1 or = 1 (blue). (**C**) γH2A.X (p-S139) IF puncta count of cells treated with different inhibitors for indicated time points. The central line is median, bottom and top edges are 25th and 75th percentiles. The whiskers show the most extreme data points, and the outliers (x) are plotted individually.

Data are representative of at least two independent experiments, n > 24,000 cells per condition. (**D**) Percentage of cleaved Caspase 3 positive cells treated with different inhibitors for 24 hours. Data are representative of at least two independent experiments, n > 24,000 cells per condition. (**E**) Bar plot shows the percent of mitotic defects among all mitotic events in a population of cells treated with DMSO or FASN inhibitors. Mitotic defects called using H2B-mTurquoise signal. At least 41 mitotic events (average: 150 events) were quantified per condition. Data are from two independent experiments, n > 8,000 per condition. (**F**) Sample microscopy images of cycling H2B-mTurquoise expressing MCF-10A cells in the presence of C75 (added at 168 min time point). Time stamp shows minutes. Scale bar: 10 µm. Arrows indicate cells undergoing mitotic failure. Data are representative of two independent experiments. (**G**) Single-cell traces of cyclin-E/A CDK activity (blue) in cycling cells treated with different inhibitors or DMSO control two hours before mitosis. Grey traces (cyclin-E/A CDK activity < 0.6 for less than 5 hours). Quantification is percentage of cells with cyclin-E/A CDK activity > 0.6 for at least 5 hours (blue). (**H**) Plot shows percentages of cells with a cyclin-E/A CDK activity > 0.6 for at least 5 hours. (**A, B, G, H**) Traces are 100 random cells. Quantification is mean percentage ± SD of two independent experiments, n > 75,000 cells per condition. (**G, H**) n > 20,000 per condition. APC/C^{Cdh1}, anaphase-promoting complex/cyclosome-Cdh1; MMS, Methyl Methanesulfonate. Inhibitor concentrations used: C75 (30µM), GSK2194069 (50µM), Cerulenin (15µM), SCDi (32µM), MMS, (500ng/ml), NCS (200ng/ml). Source data are provided as Source data file.

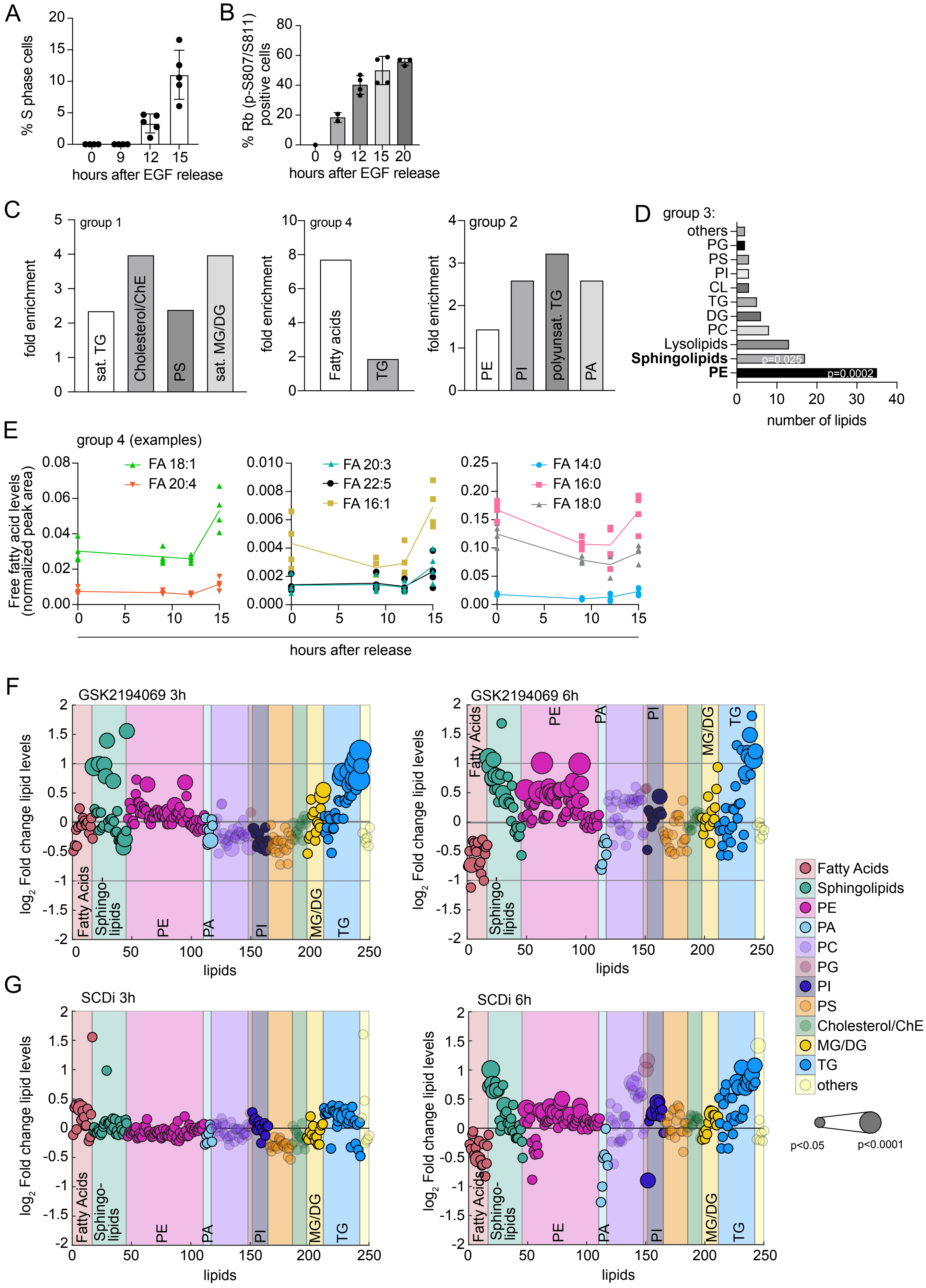
A
% S phase cells
hours after EGF release
B
% Rb (p-S807/S811) positive cells
hours after EGF release
C
group 1
fold enrichment
sat. TG
Cholesterol/ChE
PS
sat. MG/DG
group 4
Fatty acids
TG
group 2
PE
PI
polyunsat. TG
PA
D
group 3:
others
PG
PS
PI
CL
TG
DG
PC
Lysolipids
Sphingolipids
PE
p=0.025
p=0.0002
number of lipids
E
group 4 (examples)
Free fatty acid levels (normalized peak area)
FA 18:1
FA 20:4
FA 20:3
FA 22:5
FA 16:1
FA 14:0
FA 16:0
FA 18:0
hours after release
F
GSK2194069 3h
GSK2194069 6h
log2 Fold change lipid levels
lipids
G
SCDi 3h
SCDi 6h
Fatty Acids
Sphingolipids
PE
PA
PC
PG
PI
PS
Cholesterol/ChE
MG/DG
TG
others
p<0.05
p<0.0001

Fig. S3: Inhibition of fatty acid metabolism changes the lipidome. (**A**) Percentage of MCF-10A cells in S phase at different time points after EGF release. Data are from five independent experiments, shown as mean ± SD, n > 14,000 cells per condition. (**B**) Percentage of Rb (p-S807/8011) positive MCF-10A cells at different time points after EGF release. Data are from at least two independent experiments, shown as mean ± SD, n > 15,000 cells per condition. (**C**) Bar graphs show significant fold enrichment of lipid classes per lipid group from **Fig. 2B** across all 250 lipids measured. P values are calculated using Fisher's exact test. Data are from four independent experiments. (**D**) Bar graph shows the number of lipids per class in group 3. Significantly enriched lipid classes are printed in bold. Data are from four independent experiments. (**E**) Individual fatty acid examples from group 4 (**Fig. 2B**) shown as lipid concentration (normalized peak area) over time. Data are from four independent experiments. (**F, G**), Lipidome analysis of 250 lipids in MCF-10A cells treated with GSK2194069 (**F**) or SCDi (**G**) for 3 hours (left) or 6 hours (right). Values are shown as $\log_2$ fold-change relative to DMSO control treated cells. Each dot represents a lipid species, background and dots are color-coded per lipid class. Dot size indicates significance. Data are combined of four independent experiments and represented as mean. Inhibitor concentrations used unless indicated otherwise: C75 (30µM), GSK2194069 (50µM), SCDi (32µM). Source data are provided as Source data file.

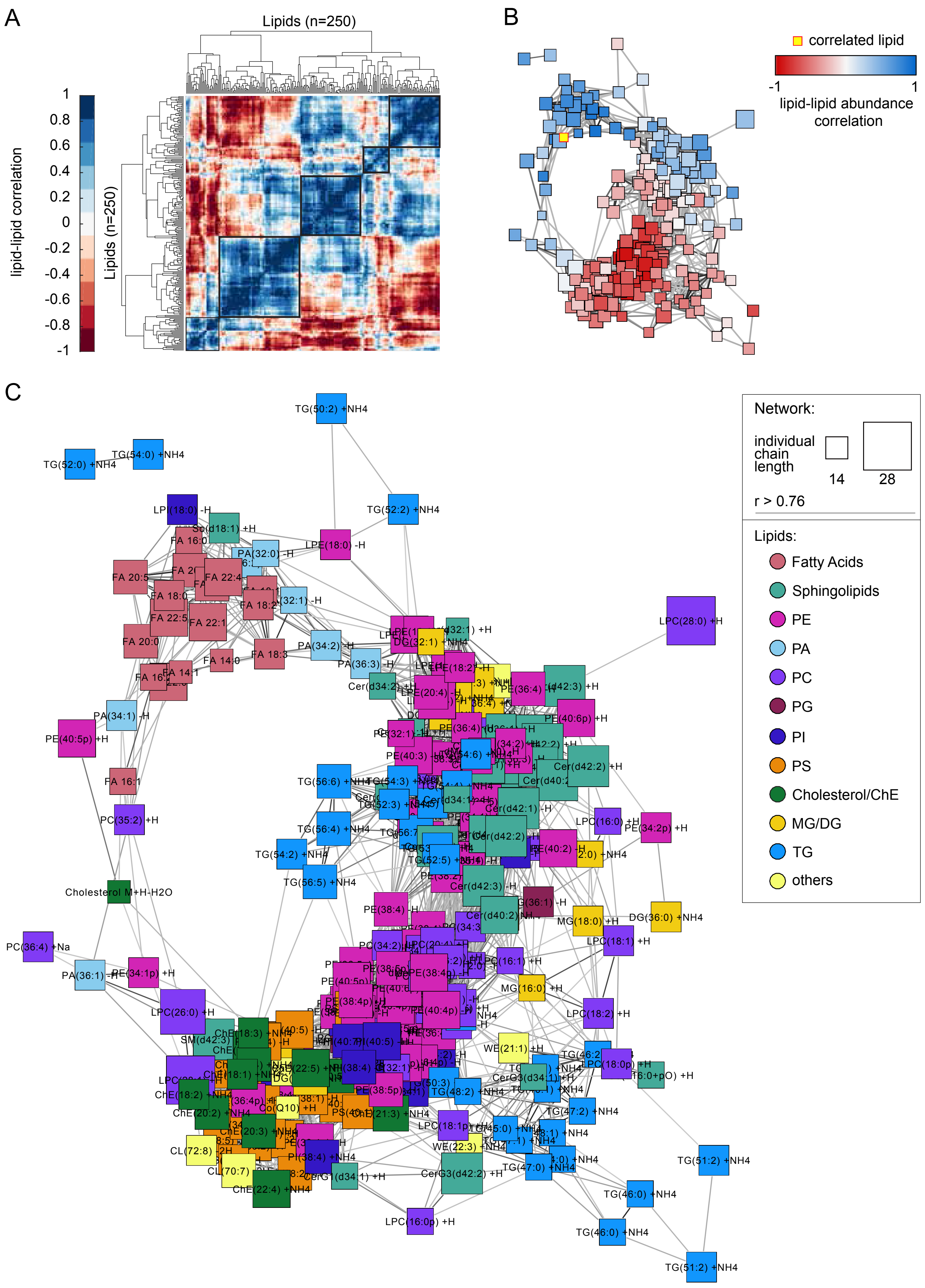

A
Lipids (n=250)
lipid-lipid correlation
1
0.8
0.6
0.4
0.2
0
-0.2
-0.4
-0.6
-0.8
-1
Lipids (n=250)
B
correlated lipid
-1
1
lipid-lipid abundance correlation
C
Network:
individual chain length
14
28
r > 0.76
Lipids:
Fatty Acids
Sphingolipids
PE
PA
PC
PG
PI
PS
Cholesterol/ChE
MG/DG
TG
others
TG(50:2) +NH4
TG(54:0) +NH4
TG(52:0) +NH4
TG(52:2) +NH4
LPE(18:0) -H
LPC(28:0) +H
PE(40:5p) +H
PC(35:2) +H
Cholesterol M+H-H2O
PC(36:4) +Na
PA(36:1) -H
LPC(26:0) +H
TG(56:6) +NH4
TG(56:4) +NH4
TG(54:2) +NH4
TG(56:5) +NH4
PE(38:4) -H
Cer(d42:2) +H
MG(18:0) +H
DG(36:0) +NH4
LPC(18:1) +H
MG(16:0) +H
LPC(18:2) +H
TG(51:2) +NH4
TG(46:0) +NH4
LPC(16:0p) +H
CerG3(d42:2) +H
ChE(22:4) +NH4

Fig. S4: The circular network of lipid coregulation. (**A**) Hierarchical clustering of lipid-lipid correlations. Rows and columns correspond to the 250 measured lipid species. Black boxes indicate clusters of strongly positively correlated lipids. Data are combined of four independent experiments and shown as mean. (**B**) Circular network color-coded by lipid correlation with fatty acid FA 14:1 (Myristoleic Acid, yellow node) abundance. (**C**) Network visualization of the positive lipid-lipid correlations. Edges are correlations of r > 0.76. Nodes are lipids color-coded by lipid class (see legend). Node size represents fatty acid chain length. Data are combined of four independent experiments.

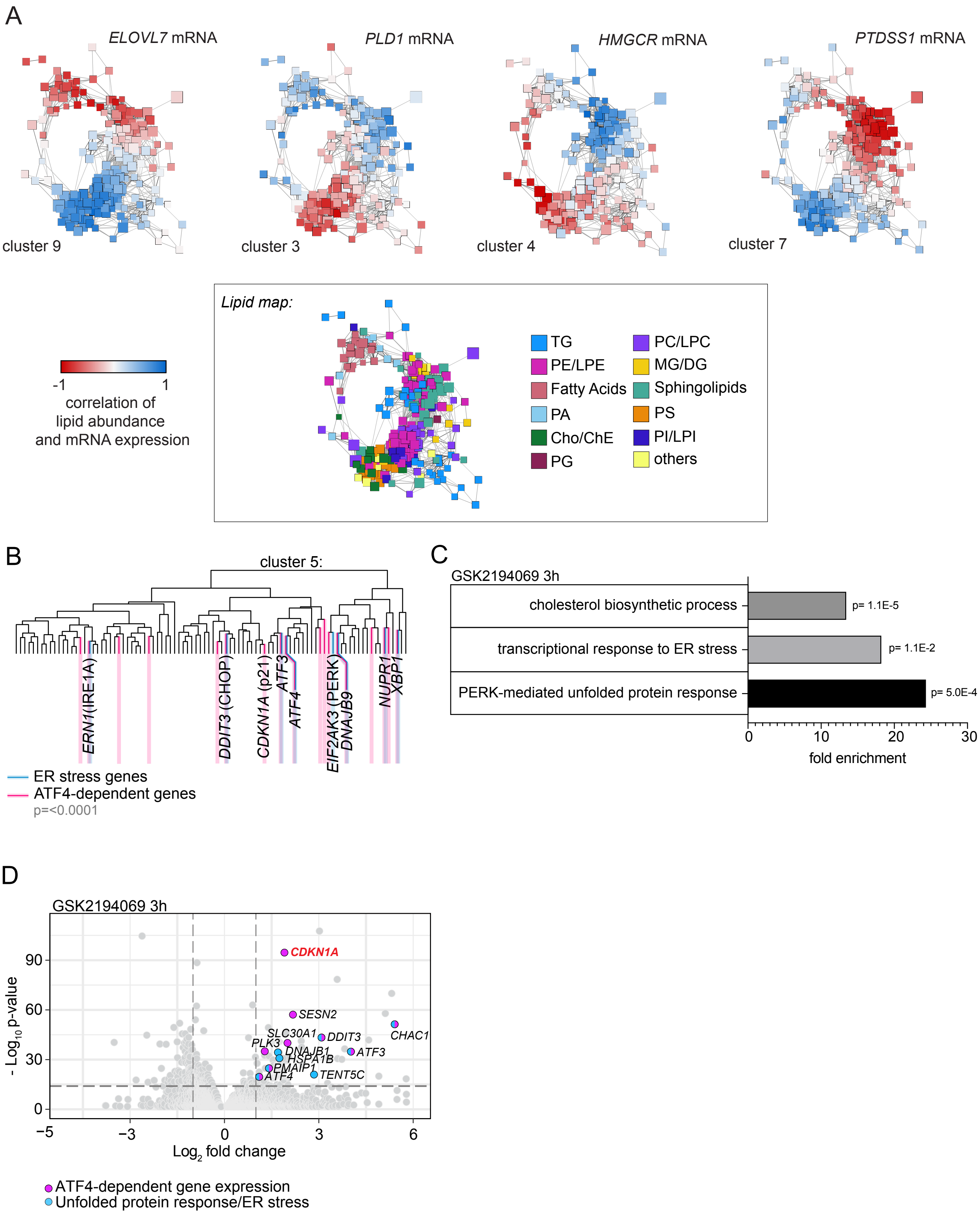

Fig. S5: Transcriptional changes upon FASN inhibition and lipidome correlations. (**A**) Correlations of *ELOVL7*, *PLD1*, *HMGCR* and *PTDSS1* mRNA levels with 250 lipids color-coded on the circular network. Legend: For orientation the lipid map shows the nodes of the network

color-coded based on different lipid classes. (**B**) Dendrogram is part of cluster 5 showing 93 genes clustered based on their correlations with 250 lipids. Magenta lines represent ATF4-dependent genes[1]. Blue lines represent genes that are associated with the ER stress pathway based on GO terms. This part of cluster 5 is significantly enriched for ATF4-dependent genes. P value is based on Fisher's exact test for enrichment analysis. (**C**) Bar plot shows fold enrichment for GO Terms (BP direct) among genes that were significantly upregulated in cells treated with GSK2194069 for 3 hours at 9 hours after EGF release (mRNA sequencing data, significant in at least three biological replicates, p.adj. <0.05, filtered for increased expression in starved cells and at least 2-fold upregulated compared to DMSO treatment). P values were calculated using EASE score (Fisher's Exact). Processes are sorted by false discovery rate (FDR)–adjusted P value. Redundant processes omitted. (**D**) Volcano plot shows differentially regulated genes after 3 hours of GSK2194069 treatment. Differentially expressed genes that meet the thresholds of $\log_2$(fold-change) | > 1, p-value < 0.014 are either highlighted in blue (genes associated with the ER stress pathway/Unfolded Protein response based on GO terms) or magenta (ATF4-dependent genes[1]) or both (blue-magenta). *CDKN1A* (p21) is highlighted in red as the ER stress-cell cycle link. Inhibitor concentration used: GSK2194069 (50µM).

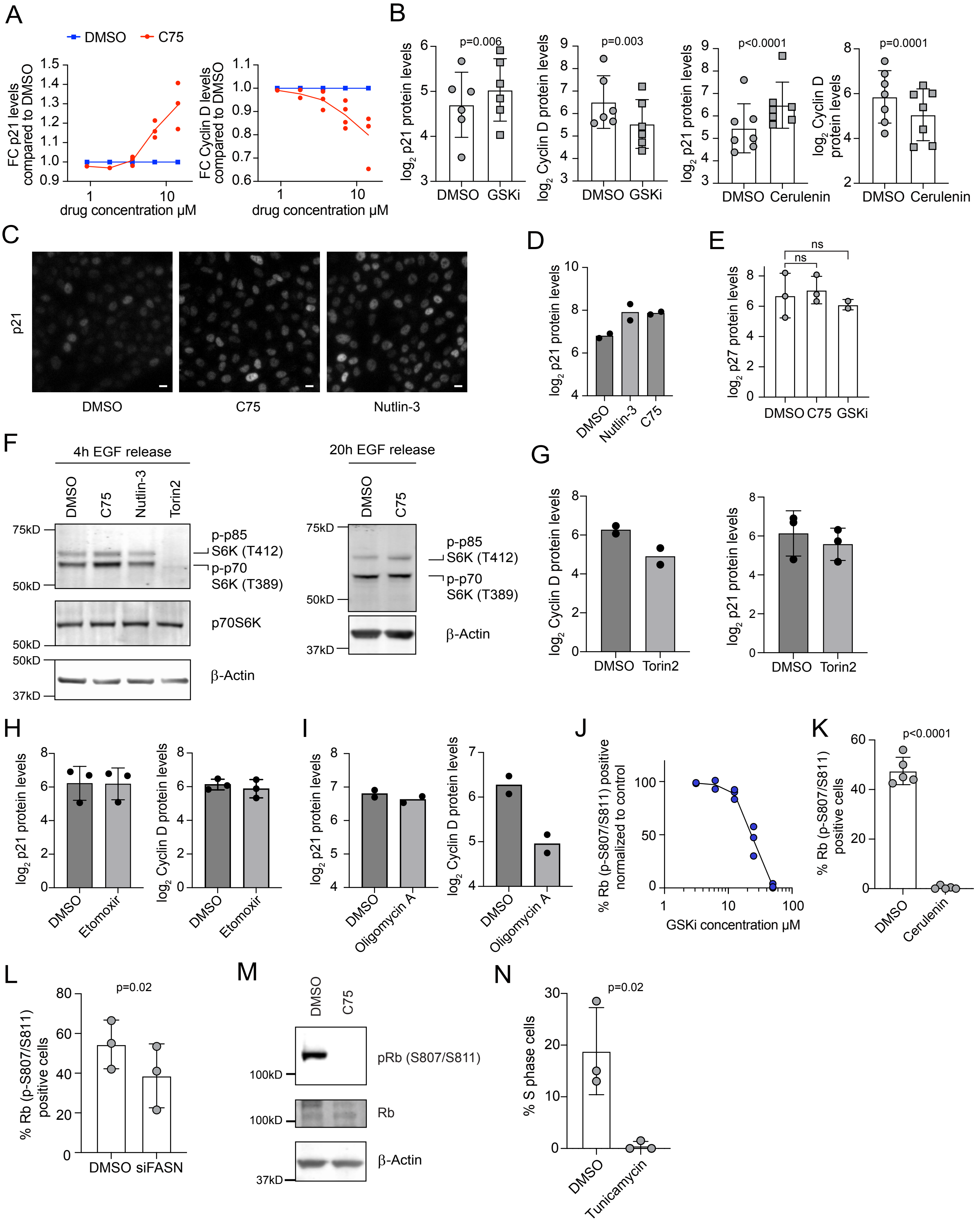

Fig. S6: p21 induced by FASN inhibition is independent of mTOR and mitochondrial function. (**A**) Fold change (FC) of p21 and Cyclin D nuclear protein levels (red) measured by immunofluorescence 4 hours after EGF release treated with increasing concentrations of C75 and

normalized to DMSO (blue). Data are from at least two independent experiments, n > 12,000 cells per condition. (**B**) p21 and Cyclin D nuclear protein levels measured by immunofluorescence 4 hours after EGF release treated with DMSO, GSK2194069, or Cerulenin. Data are from at least six independent experiments, n > 12,000 cells per condition. (**C**) Nuclear p21 levels in cells released with EGF for 4 hours treated with DMSO, C75, or Nutlin-3. Sample immunofluorescence images are shown. Scale bar: 10 μm. Data are representative of at least two independent experiments. (**D**) Quantification of (**C**). Data are from two independent experiments, n > 8,000 cells per condition. (**E**) Nuclear p27 protein levels measured by immunofluorescence 5 hours after EGF release and treated with DMSO, C75, or GSK2194069. Data are from at least two independent experiments, n > 22,000 cells per condition. (**F**) MCF-10A cells were EGF released for indicated time points and treated with different inhibitors. Blotted for p-p70 S6 Kinase (T389), p70 S6 Kinase, and β-actin. Data are representative of three independent experiments. (**G - I**) Cyclin D and p21 nuclear protein levels measured by immunofluorescence 4 hours after EGF release treated with DMSO or indicated inhibitors. Data are from at least two independent experiments, n > 8,000 cells per condition. (**J**) Dose response of percent Rb (p-S807/S811) positive cells treated with increasing concentrations of GSK2194069 normalized to DMSO measured 20 hours after EGF release. Data are from at least two independent experiments, n > 17,000 cells per condition. (**K**) Percentage of Rb (p-S807/S811) positive cells treated with DMSO or Cerulenin measured by immunofluorescence 20 hours after EGF release. Data are from five independent experiments, n > 23,000 cells per condition. (**L**) Percentage of Rb (p-S807/S811) positive cells 20 hours after EGF release transfected with control (sictrl) or siFASN measured by immunofluorescence. Data are from three independent experiments, n > 21,000 cells per condition. (**M**) EGF-released cells (20 hours), treated with DMSO or C75, and blotted for pRb (p-S807/811), Rb, and β-actin. Data are representative of three independent experiments. (**N**) Percentage of EdU positive cells 20 hours after EGF release and treated with DMSO or Tunicamycin measured by immunofluorescence. Data are from three independent experiments, n > 12,000 cells per condition. (**B, E, K, L, N**) P values calculated using two-tailed paired t test. Inhibitor concentration used: C75 (15μM), Tunicamycin (10μg/ml), GSK2194069 (50μM), Cerulenin (15μM), Nutlin-3 (10μM), Torin2 (500nM) Etomoxir (100μM), Oligomycin A (10μM). Source data are provided as Source data file.

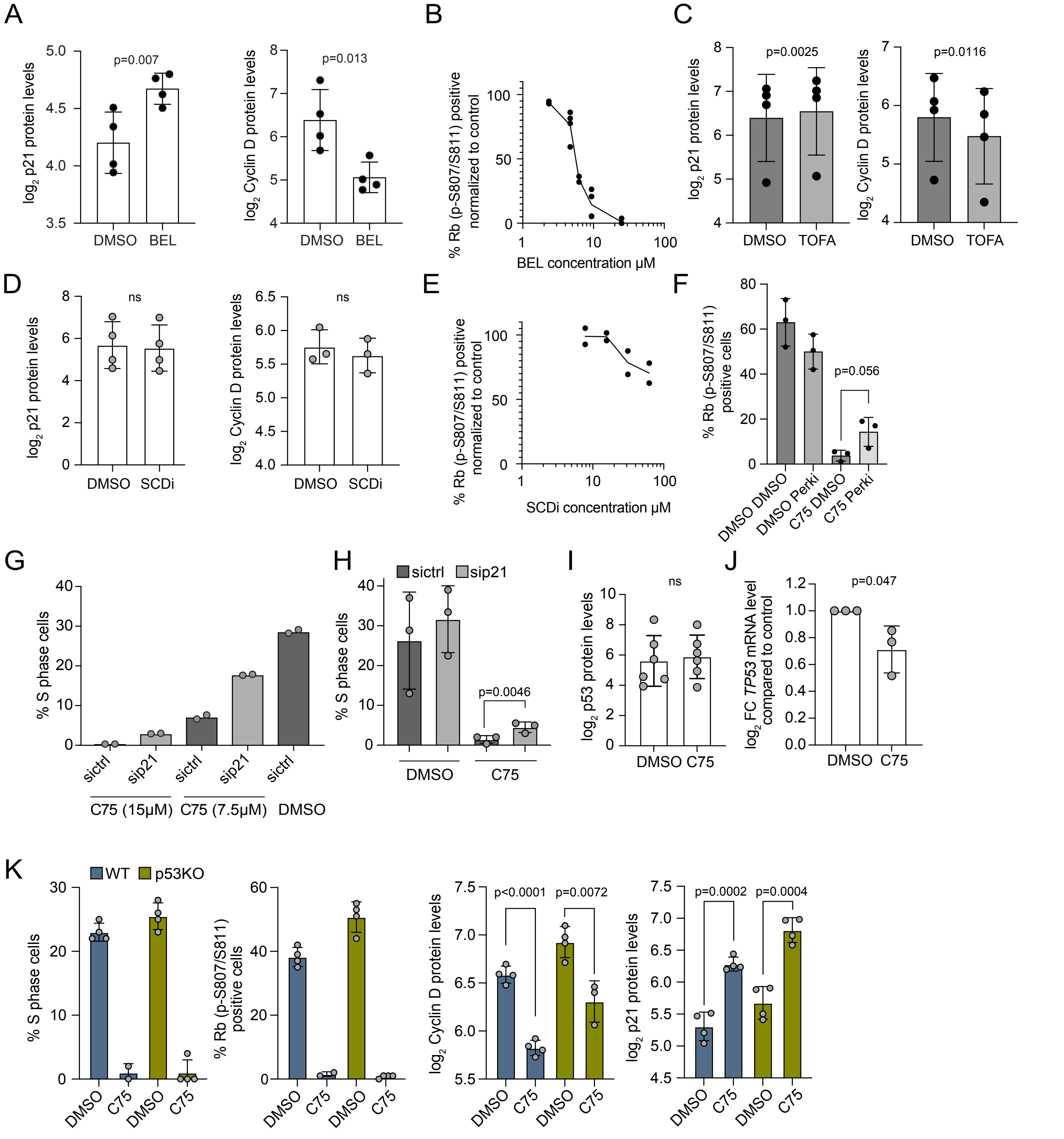

Fig. S7: FASN inhibition is partially dependent on p21 and independent of p53. (**A**) Cyclin D and p21 nuclear protein levels measured by immunofluorescence 4 hours after EGF release and treated with DMSO or BEL. Data are from four independent experiments, n > 12,000 cells per condition. (**B**) Dose response of percent Rb (p-S807/S811) positive cells treated with increasing concentrations of BEL normalized to DMSO measured 20 hours after EGF release. Data are from at least two independent experiments, n > 12,000 cells per condition. (**C**) p21 and

Cyclin D nuclear protein levels measured by immunofluorescence 4 hours after EGF release and treated with DMSO or TOFA. Data are from four independent experiments, n > 9,000 cells per condition. (**D**) p21 and Cyclin D nuclear protein levels measured by immunofluorescence 4 hours after EGF release and treated with DMSO or SCDi. Data are from at least three independent experiments, n > 12,000 cells per condition. (**E**) Dose response of percent Rb (p-S807/S811) positive cells treated with increasing concentrations of SCDi normalized to DMSO, measured 20 hours after EGF release. Data are from two independent experiments, n > 17,000 cells per condition. (**F**) Percentage of Rb (p-S807/811) positive cells measured by immunofluorescence after 20 hours of EGF release in cells treated with Perki (GSK2606414) and DMSO or C75 and corresponding DMSO control. Data are from three independent experiments, n > 15,000 cells per condition. (**G**) Percentage of EdU positive cells measured by immunofluorescence 20 hours after EGF release in cells transfected with sip21 or sicontrol (sictrl) and treated with two different concentrations of C75 (15μM and 7.5μM) or DMSO. Data are representative of at least two independent experiments, n > 21,000 cells per condition. (**H**) Percentage of EdU positive cells transfected with sictrl or sip21 and treated with DMSO or C75. Data are from three independent experiments, n > 14,000 cells per condition. (**I**) Nuclear p53 protein levels measured 5 hours after EGF release by immunofluorescence in the presence of DMSO or C75. Data are from six independent experiments, n > 16,000 cells per condition. (**J**) *TP53* mRNA expression 5 hours after EGF release measured by qRT PCR treated with C75 and normalized to DMSO. Data are from three independent experiments. (**K**) Control MCF-10A cells and p53KO cells treated with DMSO or C75 for 20 hours after EGF release to measure percentage EdU or percentage Rb (p-S807/S811) positive cells or treated for 4 hours to measure nuclear Cyclin D and p21 levels by immunofluorescence. Data are from at least two independent experiments, n > 23,000 cells per condition. (**A, C, D, F, H - K**) Mean and SD are shown. P values calculated using two-tailed Paired t test. Inhibitor concentration used unless indicated otherwise: C75 (15μM), Tunicamycin (10μg/ml), GSK2194069 (50μM), SCDi (32μM), Cerulenin (15μM), BEL (9μM), TOFA (12.5μM). Source data are provided as Source data file.

Fig. S6F

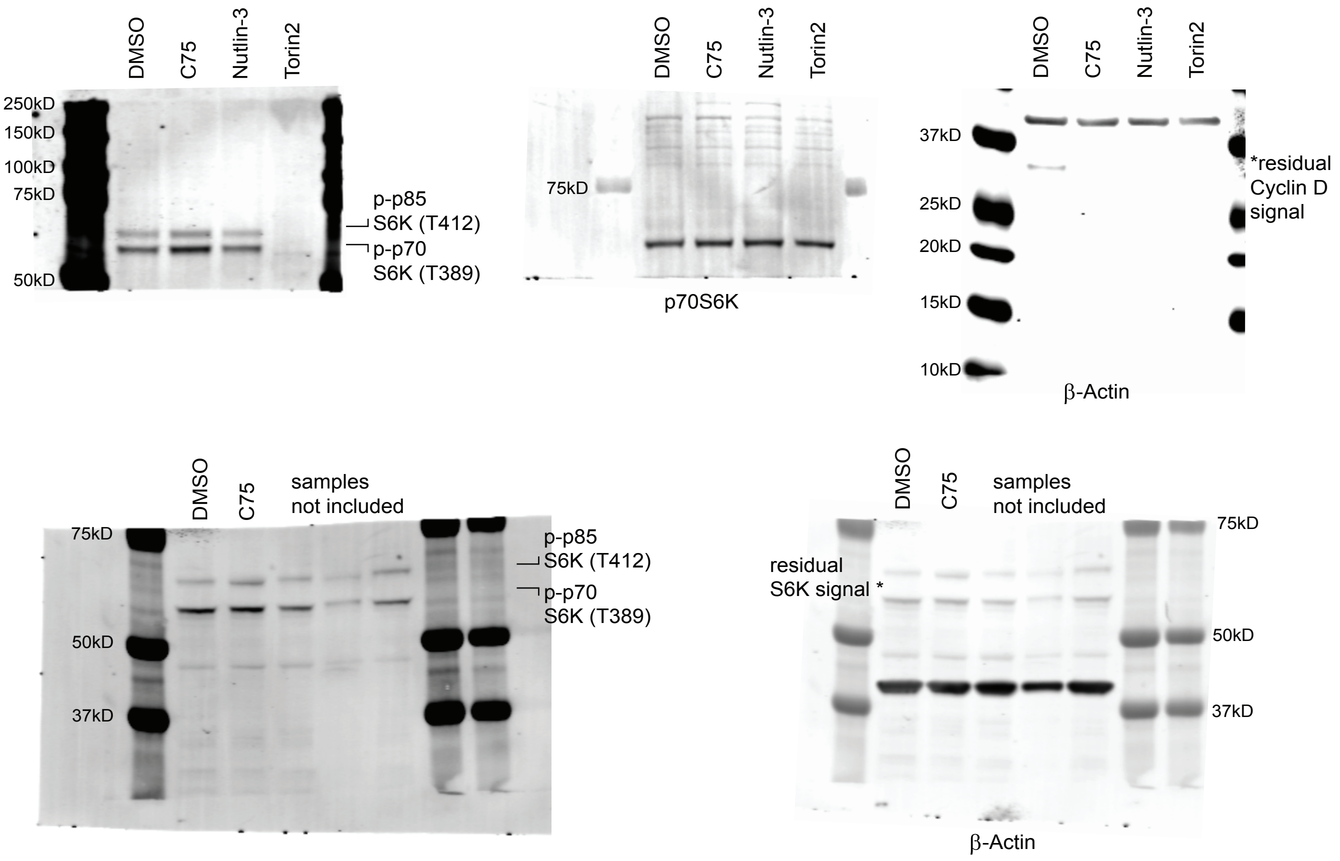

Fig. S6M

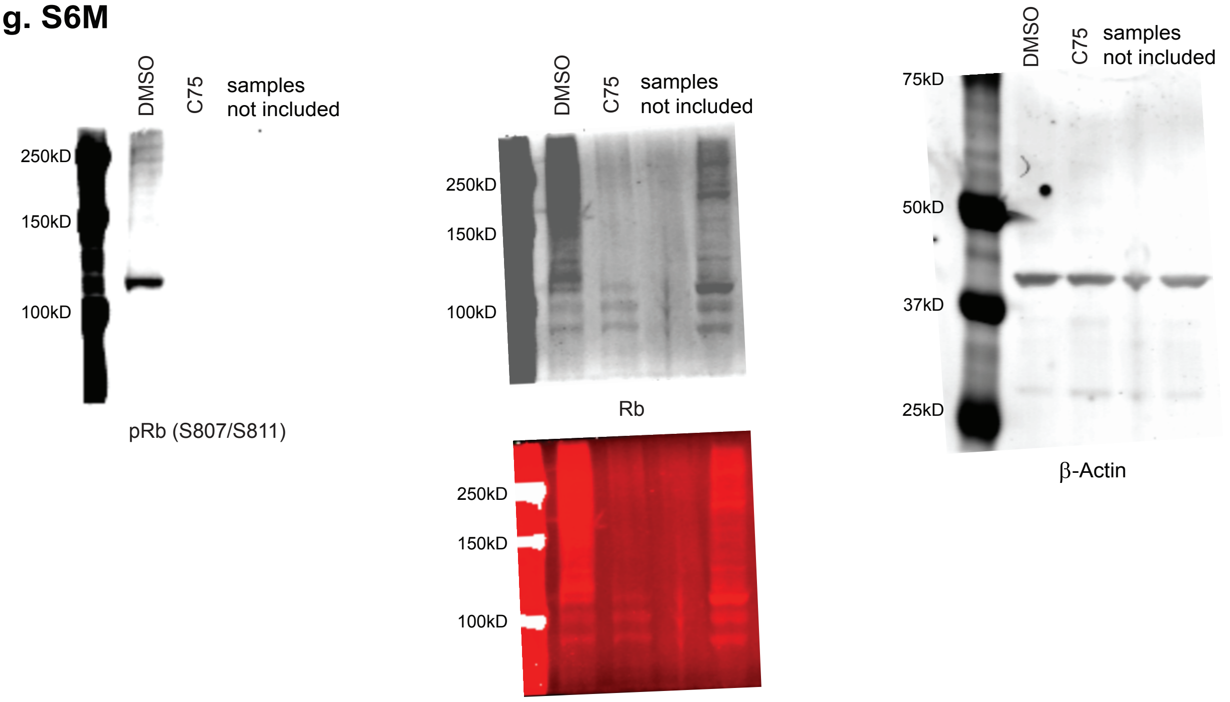

Reference:

1. Torrence, M. E. *et al.* The mTORC1-mediated activation of ATF4 promotes protein and glutathione synthesis downstream of growth signals. *eLife* **10**, e63326 (2021).

Catalog Number	Target gene	Gene ID	Sequence	PAM	Genomic Location	Accession Exons
CM-003954-01	FASN	2194	GTGTAGGCCAGTACGTAGGT	GGG	hg38|+chr17:82080374-82080396	NM_004104.4 (Exon40)
CM-003954-02	FASN	2194	CTTGATGATCAGGTCCACGG	CGG	hg38|+chr17:82079537-82079559	NM_004104.4 (Exon42)
CM-003954-03	FASN	2194	TGAGAAGGCTGCGGCCTATA	GGG	hg38|-chr17:82081649-82081671	NM_004104.4 (Exon37)
CM-003954-04	FASN	2194	CTGATCATCAAGAGCCACCA	GGG	hg38|-chr17:82079526-82079548	NM_004104.4 (Exon42)
CM-003954-05	FASN	2194	ATGGAACACCGTGCACTTGA	GGG	hg38|+chr17:82083271-82083293	NM_004104.4 (Exon32)

Supplementary Table 1: crRNA sequences

Catalog number from Horizon Discovery (column A), target gene name (column B), gene ID (column C), sequence (column D), PAM sequence (column E), genomic targeting location (column F), and accession exons (column G) are shown.

Catalog Number	Target gene	Gene accession	GI number	Sequence
J-003471-09	CDKN1A	NM_000389	17978496	CGACUGUGAUGCGCUAAUG
J-003471-10	CDKN1A	NM_000389	17978496	CCUAAUCCGCCCACAGGAA
J-003471-11	CDKN1A	NM_000389	17978496	CGUCAGAACCCAUGCGGCA
J-003471-12	CDKN1A	NM_000389	17978496	AGACCAGCAUGACAGAUUU
J-003954-11	FASN	NM_004104	41872630	UGACAUCGUCCAUUCGUUU
J-003954-12	FASN	NM_004104	41872630	GAAGCACAUUGGCAAAGUC
J-003954-13	FASN	NM_004104	41872630	GGUAUGCGACGGGAAAGUA
J-003954-14	FASN	NM_004104	41872630	CUUCCGAGAUUCCAUCCUA
D-001810-10-05	non-targeting control			GCCCUUCUCUGCAGUCAAG
D-001810-10-05	non-targeting control			UGGUUUACAUGUUGUGUG
D-001810-10-05	non-targeting control			UGGUUUACAUGUUUUCCUA
D-001810-10-05	non-targeting control			UGGUUUACAUGUUUUCUGA

Supplementary Table 2: siRNA sequences

Catalog number from Horizon Discovery (column A), target gene name (column B), gene accession (column C), GI number (column D), and sequences (column E) are shown.
