# Supplementary material for: A fast-acting lipid checkpoint in G1 prevents mitotic defects
Source: Nat Commun. 2024 Mar 18;15:2441. doi: 10.1038/s41467-024-46696-9 (PMC10948896; doi:10.1038/s41467-024-46696-9)
Supplement: Supplementary file 3 — Description of Additional Supplementary Files [file 41467_2024_46696_MOESM3_ESM.pdf]

Description of Additional Supplementary Files

File Name: Supplementary Data 1
Description: Lipidome time course analysis. Lipidome analysis of 250 different lipid species (column A). Color coded columns are four biological replicates per condition indicated. Peak intensities normalized by total signal were used for lipid quantitation and are shown in tab "Raw Lipidomics Data". "-H" or "+H" indicate if data was acquired in positive and negative ionization modes. The second tab "log2 FC time points" shows the lipid number and nomenclature (columns A and B) and log2 fold change color coded by treatments. Cell cycle time points post EGF release (columns C – E) are normalized against the unreleased time point. Treatment time points (columns G – L) are normalized against the respective control time points. Correlation values calculated between relative lipid abundance and EdU incorporation (column N), and between the relative lipid abundance and the relative abundance of fatty acid C14:1 (column O) is shown across all treatments and time points. The third tab "Nomenclature" shows the abbreviations (column A) used in Supplementary Data 1 and the text, the lipid and inhibitor names (column B), the type of lipid or inhibitor (column C), and the protein target of the inhibitor (column D).

File Name: Supplementary Data 2
Description: Lipid-mRNA correlations. Correlation values between relative lipid abundances and relative mRNA expression per gene measured in MCF-10A cells for 250 lipid species and 3739 genes differentially regulated after three hours of C75 treatment (cutoff: log2 fold change > 0.1, FDR < 0.1) shown in first tab "lipid-gene correlations". Lipids are shown (column A) and gene names are shown (row 2). Lipid-gene correlations were used to cluster the list of genes into different groups (based on clustergram (MATLAB) in second tab "gene clusters". Cluster number (column A), gene name (column B), and ATF4-dependent regulation (column C, TRUE/FALSE) based on68 are shown. Relative mRNA expression is shown in third tab "top deregulated genes" for the 3739 differentially regulated genes (column A) upon three hours of C75 treatment across all the different treatments and time points (columns B – G).

File Name: Supplementary Data 3
Description: RNA Sequencing data. Differentially, significantly expressed genes per treatment and time point based on RNA sequencing results are shown (see different tabs). Gene names (column A), base Mean (column B), log2 fold change versus the respective control (column C), Standard error value (column D), stat (Wald statistic, column B divided by column C and compared to a standard normal distribution, column E), pvalue using the two-sided Wald test (column F), padj is the p-value adjusted using Benjamini and Hochberg method (column G).
