# Supplementary material for: A fast-acting lipid checkpoint in G1 prevents mitotic defects
Source: Nat Commun. 2024 Mar 18;15:2441. doi: 10.1038/s41467-024-46696-9 (PMC10948896; doi:10.1038/s41467-024-46696-9)
Supplement: Supplementary file 7 — Reporting Summary [file 41467_2024_46696_MOESM7_ESM.pdf]

nature portfolio

Corresponding author(s): Marielle S Köberlin, Tobias Meyer

Last updated by author(s): Feb 22, 2024

Reporting Summary

Nature Portfolio wishes to improve the reproducibility of the work that we publish. This form provides structure for consistency and transparency in reporting. For further information on Nature Portfolio policies, see our Editorial Policies and the Editorial Policy Checklist.

Statistics

For all statistical analyses, confirm that the following items are present in the figure legend, table legend, main text, or Methods section.

n/a	Confirmed	
☐	☒	The exact sample size (*n*) for each experimental group/condition, given as a discrete number and unit of measurement
☐	☒	A statement on whether measurements were taken from distinct samples or whether the same sample was measured repeatedly
☐	☒	The statistical test(s) used AND whether they are one- or two-sided<br>*Only common tests should be described solely by name; describe more complex techniques in the Methods section.*
☐	☒	A description of all covariates tested
☐	☒	A description of any assumptions or corrections, such as tests of normality and adjustment for multiple comparisons
☐	☒	A full description of the statistical parameters including central tendency (e.g. means) or other basic estimates (e.g. regression coefficient) AND variation (e.g. standard deviation) or associated estimates of uncertainty (e.g. confidence intervals)
☐	☒	For null hypothesis testing, the test statistic (e.g. *F*, *t*, *r*) with confidence intervals, effect sizes, degrees of freedom and *P* value noted<br>*Give P values as exact values whenever suitable.*
☒	☐	For Bayesian analysis, information on the choice of priors and Markov chain Monte Carlo settings
☐	☒	For hierarchical and complex designs, identification of the appropriate level for tests and full reporting of outcomes
☒	☐	Estimates of effect sizes (e.g. Cohen's *d*, Pearson's *r*), indicating how they were calculated

Our web collection on statistics for biologists contains articles on many of the points above.

Software and code

Policy information about availability of computer code

Data collection	Illumina NextSeq platform, imaging: ImageXpress Micro (Confocal), MetaXpress software (Molecular Devices), ECLIPSE Ti2 inverted microscope (Nikon), Andor Zyla sCMOS camera, Vanquish HPLC online with a Q-Exactive quadrupole-orbitrap mass spectrometer (Thermo Fisher), Roche Lightcycler480 II, Odyssey Infrared Imaging System (LI-COR Biosciences).
Data analysis	ImageJ2 (Fiji) 2.3.0/1.53f, Matlab R2020a, peak integration (Skyline), LipidSearch (Thermo), Gene Ontology (https://david.ncifcrf.gov/), R-studio (1.1.463).

For manuscripts utilizing custom algorithms or software that are central to the research but not yet described in published literature, software must be made available to editors and reviewers. We strongly encourage code deposition in a community repository (e.g. GitHub). See the Nature Portfolio guidelines for submitting code & software for further information.

Data

Policy information about availability of data

All manuscripts must include a data availability statement. This statement should provide the following information, where applicable:

- Accession codes, unique identifiers, or web links for publicly available datasets
- A description of any restrictions on data availability
- For clinical datasets or third party data, please ensure that the statement adheres to our policy

The lipidomics data generated in this study is available in Supplementary Data 1. The RNA Sequencing data generated in this study have been deposited in the GEO

(Gene Expression Omnibus) database under the accession code GSE254479 (https://www.ncbi.nlm.nih.gov/geo/query/acc.cgi?acc=GSE254479). The differential gene expression data derived from the RNA Sequencing data are available in Supplementary Data 3. The lipid-mRNA correlations generated in this study based on the lipidomics and differential gene expression analysis are available in Supplementary Data 2. Databases used for data analysis include human genome assembly (GRCh38, https://www.ncbi.nlm.nih.gov/datasets/genome/GCF_000001405.26/), Gene Ontology (GO) by DAVID Bioinformatics (https://david.ncifcrf.gov/), ATF4-dependent genes taken from this publicly available RNA Sequencing dataset GSE158605 (https://www.ncbi.nlm.nih.gov/geo/query/acc.cgi?acc=GSE158605). Uncropped scans of Western Blots are available in the Source Data file and in the Supplementary Information file. Source data are provided with this paper. Raw images supporting the findings of this study are available from the corresponding authors upon reasonable request.

Research involving human participants, their data, or biological material

Policy information about studies with human participants or human data. See also policy information about sex, gender (identity/presentation), and sexual orientation and race, ethnicity and racism.

Reporting on sex and gender	no human research participants
Reporting on race, ethnicity, or other socially relevant groupings	no human research participants
Population characteristics	no human research participants
Recruitment	no human research participants
Ethics oversight	no human research participants

Note that full information on the approval of the study protocol must also be provided in the manuscript.

Field-specific reporting

Please select the one below that is the best fit for your research. If you are not sure, read the appropriate sections before making your selection.

☒ Life sciences ☐ Behavioural & social sciences ☐ Ecological, evolutionary & environmental sciences

For a reference copy of the document with all sections, see nature.com/documents/nr-reporting-summary-flat.pdf

Life sciences study design

All studies must disclose on these points even when the disclosure is negative.

Sample size	We analyze 9 fields of view per well and quantify about 80-90% of all cells in a well (n = 8,000 – 17,000 cells). Each biological replicate is comprised of 2 wells (18 different imaging sites). This is sample size was chosen to accurately represent the population of cells and allow for reliable statistical analyses.
Data exclusions	For asynchronously cycling cells certain cell populations were analyzed (stated in text). For Gene Ontology analyses redundant processes were omitted.
Replication	Each measurement was performed at least in technical duplicates and biological duplicates (mostly triplicates or quadruplicates as stated in text). All attempts at replication of results in specific conditions were successful.
Randomization	Cells were always plated randomly into different wells.
Blinding	Blinding was not relevant to this study because data analysis and data acquisition was fully automatized using high throughput analysis pipelines.

Reporting for specific materials, systems and methods

We require information from authors about some types of materials, experimental systems and methods used in many studies. Here, indicate whether each material, system or method listed is relevant to your study. If you are not sure if a list item applies to your research, read the appropriate section before selecting a response.

Materials & experimental systems

n/a	Involved in the study
☐	☒ Antibodies
☐	☒ Eukaryotic cell lines
☒	☐ Palaeontology and archaeology
☒	☐ Animals and other organisms
☒	☐ Clinical data
☒	☐ Dual use research of concern
☒	☐ Plants

Methods

n/a	Involved in the study
☒	☐ ChIP-seq
☒	☐ Flow cytometry
☒	☐ MRI-based neuroimaging

Antibodies

Antibodies used

Rb (p-807/811) (#8516, (IF: 1:2500, Western blot (WB): 1:1000)), Rb (#9309, IF: 1:1000), p21 (#2947, WB: 1:1000), p27 (#3686, IF: 1:1600), p53 (#2527, IF: 1:1600), gH2A.X (p-S139) (#2577, IF: 1:500), p70 S6Kinase (p-T389) (#9234, WB: 1:200), b-Actin (#8457, WB: 1:2000), (all Cell Signaling Technology); Rb (#554136, WB: 1:250), p21 (Clone SXM30, mouse, #556431, IF: 1:250, both BD Biosciences); Cyclin D1(SP4) (#MA5-16356, IF and WB: 1:500, Thermo Scientific); p70 S6Kinase (H-9, #sc-8418, WB: 1:200), FASN (G-11, #sc-48357, IF: 1:200) (both Santa Cruz Biotechnology).

Validation

Validated through siRNA knockdown or absence in starved cells, and localization using immunofluorescence: Rb (p-807/811) (#8516), Rb (#9309), Rb (#554136), Cyclin D1(SP4) (#MA5-16356), p21 (#2947), validated in Chung et al and Liu et al (cited for immunofluorescence in at least 4 other publications). Antibody validated using DNA damage reagents and localization with immunofluorescence: gH2A.X (p-S139) (#2577), also validated in Liu et al and Yang et al. Antibody validated using an mTOR inhibitor and localization: p70 S6Kinase (p-T389). Antibodies validated using Western blot band size: p70 S6Kinase (#sc-8418), b-Actin (#8457. Cited in 227 publications; and 1125 publications (144 for Western Blots with human samples) respectively. Antibodies validated using siRNA knockdown: p21 (Clone SXM30, mouse, # 556431), FASN (G-11, #sc-48357, cited in 73 publications).

Eukaryotic cell lines

Policy information about cell lines and Sex and Gender in Research

Cell line source(s)

MCF-10A cells (ATCC, CRL-10317), hTERT-RPE1 cells (ATCC, CRL-4000), MCF-10A p53—/—cells were obtained from Horizon Discovery (HD 101-005).

Authentication

All cell lines were validated by the companies using STR profiling. MCF-10A cells have 5296 citations and hTERT-RPE1 have 2332 citations.

Mycoplasma contamination

All cell lines were tested negative for mycoplasma contamination using PCR and/or Hoechst staining regularly.

Commonly misidentified lines
(See ICLAC register)

No commonly misidentified lines used.

Plants

Seed stocks

n/a

Novel plant genotypes

n/a

Authentication

n/a
